# Severe Elimination Disorders and Normal Intelligence in a Case of *MAP1B* Related Syndrome: A Case Report

**DOI:** 10.3390/genes16080870

**Published:** 2025-07-24

**Authors:** Aniel Jessica Leticia Brambila-Tapia, María Teresa Magaña-Torres, Luis E. Figuera, María Guadalupe Domínguez-Quezada, Thania Alejandra Aguayo-Orozco, Jesua Iván Guzmán-González, Hugo Ceja, Ingrid Patricia Dávalos-Rodríguez

**Affiliations:** 1 Departamento de Psicología Básica, Centro Universitario de Ciencias de la Salud (CUCS), Universidad de Guadalajara, Guadalajara 44340, Mexico; jesua.guzman@academicos.udg.mx; 2 División de Genética, Centro de Investigación Biomédica de Occidente (CIBO), Instituto Mexicano del Seguro Social (IMSS), Guadalajara 44340, Mexico; maganamt@gmail.com (M.T.M.-T.); luis.figuera@academicos.udg.mx (L.E.F.); madq67@yahoo.com.mx (M.G.D.-Q.); thaguayo@gmail.com (T.A.A.-O.); 3 Servicio de Neuropediatría, Hospital Civil de Guadalajara Fray Antonio Alcalde, Guadalajara 44280, Mexico; hceja@hcg.gob.mx; 4 Departamento de Biología Molecular y Genómica, Doctorado en Genética Humana, Centro Universitario de Ciencias de la Salud (CUCS), Universidad de Guadalajara, Guadalajara 44340, Mexico

**Keywords:** MAP1B, loss-of-function variants, elimination disorders, incontinence, microcephaly, short stature

## Abstract

Pathogenic variants in the *MAP1B* gene have been associated with neurological impairment, including intellectual disability, attention-deficit/hyperactivity disorder (ADHD), autism spectrum disorder, brain malformations, cognitive hearing loss, short stature, and dysmorphic features. However, few cases with detailed clinical characterization have been reported. We describe a 12-year-old boy carrying a loss-of-function *MAP1B* variant, presenting with severe elimination disorders despite normal intelligence. He was referred to the genetics service due to persistent elimination issues, including daytime urinary incontinence, nocturnal enuresis, and fecal incontinence. He had normal motor and cognitive development, with an IQ of 99; however, he also presented with ADHD, short stature, microcephaly, and myopia. Brain MRI revealed bilaterial subependymal periventricular nodular heterotopia (PVNH). Audiometry showed normal bilateral hearing. Testing fragile X syndrome (FXS) and karyotype analyses yielded normal results. Whole exome sequencing (WES) revealed a nonsense pathogenic variant in MAP1B (c.895 C>T; p.Arg299*). No other family members showed a similar phenotype; however, a great-uncle and a great-aunt had a history of nocturnal enuresis until age 10. The patient’s deceased mother had short stature and psychiatric disorders, and a history of consanguinity was reported on the maternal side. This case broadens the phenotypic spectrum associated with *MAP1B* syndrome, suggesting that elimination disorder, frequently reported in FXS, should also be evaluated in *MAP1B* pathogenic variant carriers. In addition, the presence of short stature also appears to be part of the syndrome.

## 1. Introduction

Loss-of-function (LoF) variants in the *MAP1B* gene (microtubule associated protein 1B; OMIM: *157129) in the heterozygous state have been associated with a range of neurodevelopmental phenotypes, including intellectual disability, attention-deficit/hyperactivity disorder (ADHD), autism spectrum disorder, brain malformations, and dysmorphic features [1]. Among brain malformations, the periventricular nodular heterotopia (PVNH) has been the most consistently reported. Additional findings include reduced white matter volume and agenesis of the corpus callosum [2]. Other reported features include short stature, microcephaly, focal epilepsy, and cognitive hearing loss [1]. Despite the phenotypic variability observed among reported cases of *MAP1B* LoF variants [1,2,3]. PVNH remains one of the most consistent findings. Incomplete penetrance has been proposed to explain some apparently sporadic presentations [3]. To date, most reported LoF variants are inherited and are located within exon 5 of the *MAP1B* gene [1,2,3].

The wide phenotypic spectrum is likely explained by the essential role of MAP1B in neuronal migration through the microtubule cytoskeleton [4], a function it performs in combination with the Fragile X mental retardation protein (FMRP) [5]. Additionally, MAP1B interacts with other neurodevelopment regulators such as BCL11A and KIRREL3 [6,7], and may function as a signaling protein regulating molecular pathways via guanine exchange factors, adaptor proteins, and other elements, potentially contributing to adult brain function [8]. The disruption of these roles can lead to neurological diseases.

It has been shown that microtubules are one of the major components of cytoskeleton which are composed of alpha and beta tubulin heterodimers; these are extremely dynamic structures, and their dynamic nature is essential for the neuronal remodeling, migration, and proliferation, as well as for other processes of neural development. Therefore, tubulin mutations have been associated with malformations of cortical development [9]. Likewise, microtubule-associated proteins have also been related to neuronal development; for instance, proteins such as the microtubule-associated protein 2c (MAP2c) or the microtubule motor protein kinesin-1 are proteins related to neurite formation [10,11]. In addition, microtubules and their associated proteins have been related to many neuronal development processes including axon elongation and branching, dendritogenesis, and synapse formation. Among the proteins that interact with microtubules, and which have demonstrated to have specific functions in neuronal development, are cytoplasmic linker proteins (CLIPS), kinesins, the microtubule serving proteins, and the microtubule-lattice binding proteins, which include the MAPs proteins, such as MAP1A, MAP1B, and Tau proteins [10,12]. Among these, specifically, MAP1B has been associated with microtubule growth speed in the proximal and distal axon shaft, suggesting that MAP1B may function as a regulator of microtubule dynamics during axon outgrowth [13]. In addition, it has been shown that MAP1B knockout mice exhibit several defects, including higher collateral axon branching, improper growth cone turning, as well as abnormalities in synaptic vesicle fusion and synaptic plasticity [14,15].

Elimination disorders—including urinary incontinence, nocturnal enuresis, and fecal incontinence—have been reported at significantly higher rates in individuals with fragile X syndrome (FXS) compared to age-matched controls (59.1% vs. 4.8%) [16]. Interestingly, these symptoms have also been described in female FXS cases [17], despite FXS being more common in males.

In this report, we present the case of a boy with a *MAP1B* LoF variant who exhibits severe elimination disorders in the context of otherwise normal intelligence, along with microcephaly, short stature, and ADHD. This case contributes to the clinical delineation of *MAP1B*-related syndromes and highlights the relevance of assessing elimination disorders in affected individuals.

## 2. Case Report

The proband is a 12-year-old male who was referred to a genetics service of a tertiary care hospital in Mexico due to persistent elimination disorders, including daytime urinary incontinence, nocturnal enuresis, and fecal incontinence.

**Figure 1:**
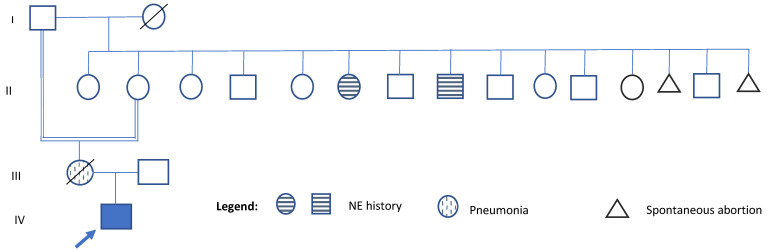
Pedigree of the family. Generation numbers are indicated on the left side of the pedigree. Within each generation, individuals are numbered from left to right. A consanguineous relationship is noted between the proband’s grandmother and her great-grandfather (individuals I-1 and II-2). The three individuals marked with a grid pattern represent family members who share clinical features with the proband. These include the proband’s mother, who had short stature (individual III-1), and two great-relatives with a history of nocturnal enuresis (NE) (individual II-6 and II-8). IV- 1 indicates the proband.

He was born at 38 weeks of gestation via cesarean section to a 25-year-old primigravida mother (G1P1). No information is available about the father. Birth weight was 2.1 kg (<3rd percentile), length was 49 cm (25th percentile), while head circumference was not recorded. Regarding family history, the patient’s mother, who died at age 31 from pneumonia, also suffered from lymphoma, systemic lupus erythematosus, sporadic seizures, a 9-month period of pulmonary insufficiency, short stature, borderline personality disorder, and multiple suicide attempts. She was the product of an incestuous relationship between her grandfather and her mother (see Figure 1). Her cognitive and motor development were reported as normal. On his maternal side, two great-uncle and one great-aunt had a history of nocturnal enuresis until the age of 10 years (see Figure 1). No other relatives with elimination disorders, intellectual disability, or short stature were reported. The health history and identify of the father remain unknown.

The patient has been raised by his grandmother, who reports that the incontinence was first identified at age 4. The symptoms included daytime incontinence, nocturnal enuresis, and fecal incontinence. These are partially managed through behavioral strategies, such as scheduled bathroom visits during the day and before bedtime, although the child reports no urge to urinate or defecate. Despite these strategies, he frequently soils his clothes and must change and wash them almost daily. He was diagnosed with ADHD at age 7, microcephaly at age 3, and myopia at age 9. The ADHD is currently treated with risperidone, methylphenidate, fluoxetine, and aripiprazole.

Regarding development milestones, it is unclear when he began to speak. However, his great-grandmother reports that he spoke his first words around 12 months of age and walked at 19 months. He has never achieved sphincters control. Although he reports being able to feel the urge to urinate, he is unable to control it, and he does not perceive the need to defecate.

**Figure 2:**
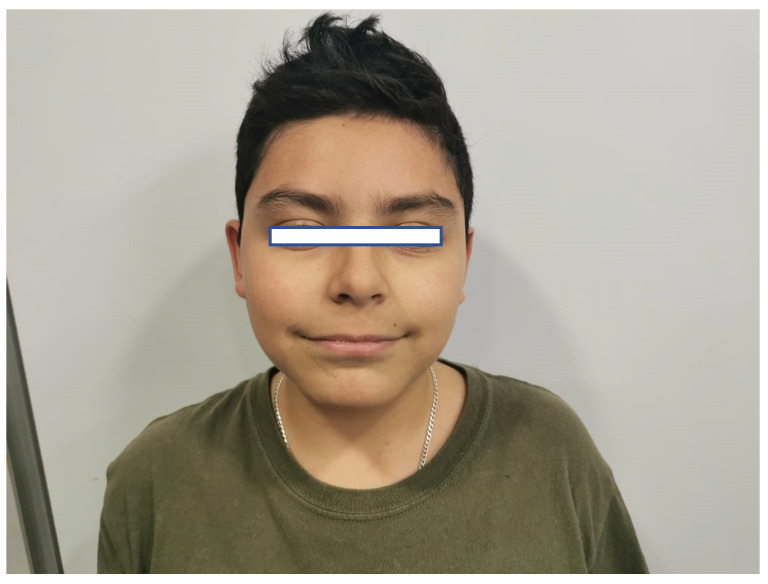
Facial features of the patient. **Figure legend:** The patient’s face, with narrow forehead, long philtrum, large mouth, and thin lips.

On physical examination, no striking facial dysmorphisms features were observed. However, mild dysmorphic features were present, including a round face with brushy arched eyebrows, mild synophrys, a narrow forehead, large almond-shaped eyes with long eyelashes, a long philtrum with poorly defined pillars, and a large mouth with thin lips (Figure 2). His hands and feet appeared normal. Anthropometric measurements include a weight of 48 kg (80th–90th percentile), height of 138 cm (<3rd percentile; near the cutoff of 139 cm), and a head circumference of 50.5 cm (<3rd percentile; near the cutoff of 50.9 cm).

**Figure 3:**
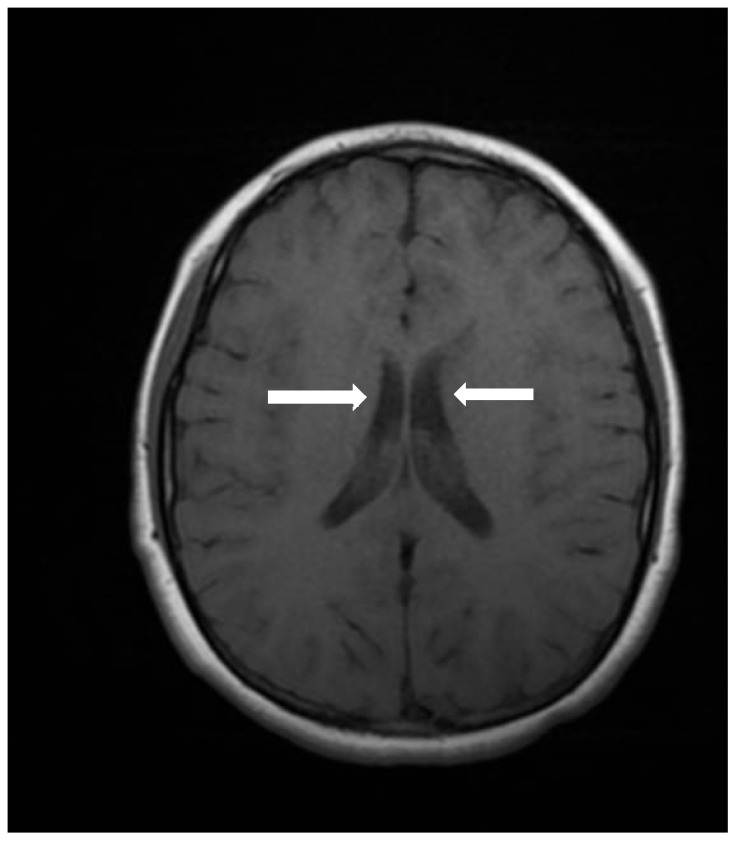

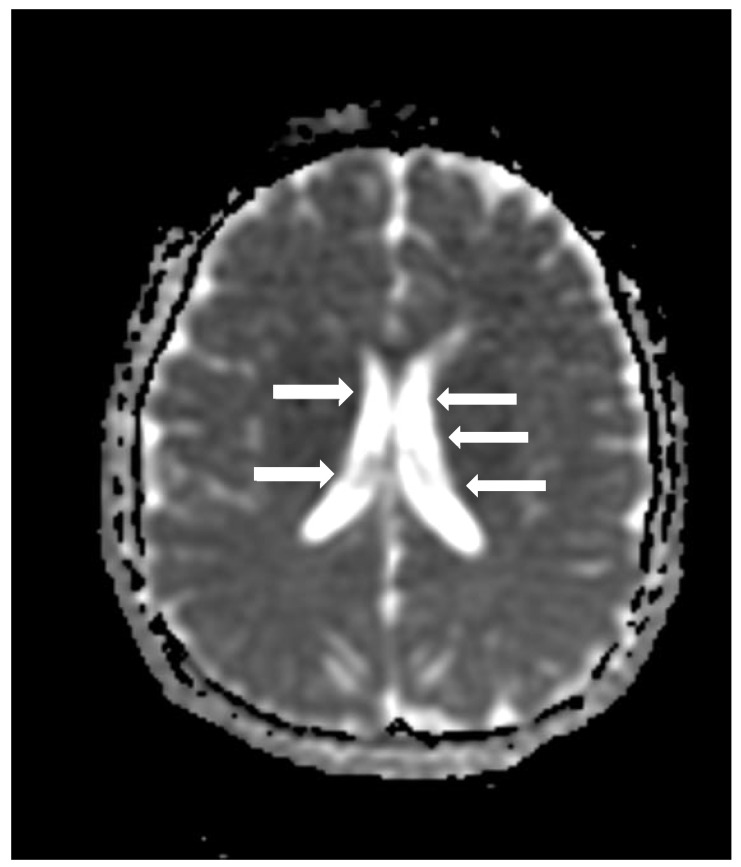
An axial image of the MRI of the skull showing PVNH in both lateral ventricles. **Figure legend:** white arrows indicate areas of PVNH, visible as discrete ripples of gray matter adjacent to lateral ventricles.

Cranial computed tomography showed a deviated nasal septum with a rightward convexity. Brain magnetic resonance imaging (MRI) with contrast revealed bilateral subependymal periventricular nodular heterotopia (PVNH) at the level of the temporal horns, with no post-contrast enhancement (Figure 3). Additionally, a right choroid plexus cyst was observed in the temporal horn. Audiometry revealed normal bilateral hearing. Neurological examinations showed only mild immaturity in fine motor coordination.

The Shipley-2 intelligence test, which evaluates verbal and non-verbal cognitive abilities [18], indicated normal cognitive performance with an IQ of 99 (56th percentile), consistent with average intelligence. The patient is currently in the first year of high school and maintains average academic performance.

Genetic testing included a standard karyotype, which was normal (46, XY [16 metaphases]), and a molecular study for FXS, which was also normal (21 CGG repeats; normal range < 45). Whole exome sequencing (WES) was performed with a deepness of 50x by the Gencell Mexico Company; the vcf files were then analyzed using the Franklin website [19] and revealed a heterozygous LoF nonsense (PVS1 classification according to the ACMG criteria) variant in exon 5 of *MAP1B* (NM_005909.5, c.895 C>T, p.Arg299*), resulting in a premature stop codon and truncated protein. No other clearly pathogenic variants were observed in the WES analysis

## 3. Discussion

We present the case of a 12-year-old boy exhibiting severe elimination disorders, microcephaly, short stature, ADHD, PVNH, and normal intelligence. These clinical features are consistent with previously reported cases involving heterozygous LoF variants in the *MAP1* gene. However, elimination disorders have not been previously associated with *MAP1B* variants. Interestingly, elimination disorders have been found in individuals with FXS, and the FMRP is known to interact with MAP1B in neuronal migration [4]. Additionally, *MAP1B*-related-syndromes and FXS (OMIM #300624), include intellectual disability, PVNH, ADHD, autism, and seizures. Therefore, elimination disorders—such as daytime urinary incontinence, nocturnal enuresis, and fecal incontinence—could represent another overlapping feature between both pathologies, potentially linked to abnormal neuronal migration and brain development, as observed in *MAP1B* knockout mice [8].

PVNH, defined as a malformation of cortical development caused by the failure of neurons to migrate to the cerebral cortex, resulting in heterotopic nodules adjacent to the lateral ventricles, is a hallmark feature of MAP1B-related syndromes [20]. LoF variants in *MAP1B* are also associated with other neurodevelopmental anomalies, such as reduced white matter volume and corpus callosum [2]. However, individuals with *MAP1B* LoF variants exhibit a wide range of phenotypic variability, with some cases showing normal intelligence and the absence of neurological symptoms [3]. Despite this variability, PVNH appears to be the most consistent neuroanatomical finding [1,2,3].

The relationship between the failure in neuronal migration and the elimination disorders could be explained by the lack of the correct development of the cortex areas in charge of rectal and anal responses, in the case of fecal incontinence, which have been mapped in the medial primary motor cortices, the supplementary motor area, the bilateral putamen and cerebellum, and the supramarginal gyrus and visual areas [21]. In the case of urination regulation, three brain areas have been identified: the pontine micturition center (PMC), the locus coeruleus, and the medial prefrontal cortex [22]. Therefore, a deficit in the correct neuronal migration to these brain areas or to the peripheric tissues, including the correct innervation of the anal and rectal areas and of the bladder and urethral sphincter, could produce fecal and urinary incontinence. However, in order to determine the specific areas affected in this case, complex functional studies of cerebral mapping and tissues biopsies are needed.

In our case, elimination disorders could be attributed to impaired neuronal migration and potentially to a genetic predisposition, given that a great-uncle and a great-aunt reportedly had nocturnal enuresis until the age of 10. This genetic susceptibility might be enhanced by consanguinity in the family. However, along with the mutation reported, the presence of severe elimination disorders could also be attributed to psychological causes, considering the psychiatric history of the mother. In addition, the patient’s mother may have carried the same variant, as suggested by her history of short stature and psychiatric symptoms, although there is no record of intellectual disability, microcephaly, or elimination disorders. Notably, *MAP1B* LoF variants have been linked to milder neurocognitive impairments, such as dyslexia or dyscalculia [2], as well as psychiatric behavioral conditions including autistic spectrum disorders (ASD) or obsessive-compulsive disorders (OCD), often accompanied by intellectual disability [2].

Although our patient does not have intellectual disability, he has ADHD, which has been reported in individuals with *MAP1B*-related syndrome. This, in combination with microcephaly and PVNH, supports the hypothesis of a neurodevelopmental basis for his severe elimination disorders, which, as noted, are also seen in FXS.

The severity of the elimination disorders may also be related to location of the variant: a nonsense variant at amino acid position 299 (p.Ar299*, full-length protein of 2468 amino acids) in exon 5 of *MAP1B.* This is one of the most proximal pathogenic LoF variants reported to date, with only two known variants occurring closer to the N-terminal: (1) p.Leu274Cyfs* 4 (c.818delC), associated with a truncated protein but no neurological symptoms [2]; and (2) p.Phe294fs (c.881del), reported in ClinVar database p.Phe294fs (variation ID: 3024282), also produced by a deletion (c.881del); this was related to hearing loss and PVNH.

In addition, we found that the mutation detected in this case has also been reported in the ClinVar database (variation ID: 2574245), referred to as a *de novo* variant in an individual with MAP1B-related neurodevelopmental disorder and brain malformations, without additional clinical information, which suggests that his mutation could be located in a hotspot. In this regard, it has been shown that hotspots are produced depending on the DNA sequence and structure, are subject to selection, and can occur due to cellular processes such as DNA replication and repair, meiotic recombination, and immunoglobulin specification. Hotspots have been linked to evolutionary and diseases mechanisms; therefore, their study and identification could help to gain knowledge about preventative and therapeutic approaches to genetic diseases [23].

It is plausible that variants located closer to the 5’end of the gene result in more truncated proteins with fewer functional transcripts, especially considering that *MAP1B* gene encodes for six different transcripts, three of them including the exon 5 [24,25]. Exon 5, the longest exon in the gene, encodes for three critical protein domains: the actin-binding domain (AB), the microtubule-binding domain (MTB), and the microtubule-assembly helping domain (MTA). The variant observed in our case lies within the AB domain [2], potentially disrupting key functions in cytoskeletal dynamics during neurodevelopment [1,2,3]. Nevertheless, to examine the specific effect of this pathogenic variant, functional assays should be performed. In addition, to examine the effects of MAP1B mutations depending on their locations, complex molecular assays are needed, such as saturation mutagenesis studies or in silico AI-fueled prediction models. These would be helpful for understanding the region-specific mutations effects [26]. In addition, it has been shown that the genomic background of each person can modify the phenotypic variability of the same pathogenic variant [27]; therefore, variable expression of MAP1B pathogenic variants, including the presence of severe eliminations disorders in this case, could also be due to the genomic background of the patient, particularly considering the presence of consanguinity.

The main limitation of this report is the absence of parental DNA, which precludes the confirmation of the variant’s origin (de novo or inherited). Additionally, the father’s medical and family history remain unknown, further limiting genotype–phenotype correlation. In addition, the absence of studies such as the Whole Genome Sequencing (WGS) impedes us from determining additional genetic variations that could be modifying the patient’s phenotype. Despite these limitations, this case expands the phenotypic spectrum related to *MAP1B* LoF variants by highlighting severe elimination disorders as a possible clinical feature, even in individuals with preserved cognitive function. It also reinforces the association of short stature with this syndrome, marking only the second reported case with this trait [1].

In conclusion, we described the case of a 12 year-old boy harboring a LoF variant in *MAP1B*, detected through WES. The main reason for the clinical evaluation was the presence of severe elimination disorders. Additional findings included microcephaly, ADHD, short stature, PVHN, and normal intelligence. While other family members did not present with the full phenotype, parental information was very limited. Consanguinity may have contributed to the phenotypic expression observed. This report supports the inclusion of elimination disorders in the clinical spectrum of MAP1B-related neurodevelopmental disorders.

## Data Availability

Data available on request due to restriction (privacy of the patient).
